# Maternal Visceral Fat in Prediction of Gestational Diabetes Mellitus

**DOI:** 10.3390/jcm13020493

**Published:** 2024-01-16

**Authors:** Jirat Detsrisuwan, Suchaya Luewan, Supatra Sirichotiyakul, Theera Tongsong

**Affiliations:** Department of Obstetrics and Gynecology, Faculty of Medicine, Chiang Mai University, Chiang Mai 50200, Thailand; jirat_det@cmu.ac.th (J.D.); supatra.s@cmu.ac.th (S.S.); theera.t@cmu.ac.th (T.T.)

**Keywords:** adipose tissue, visceral fat, gestational diabetes mellitus, ultrasound

## Abstract

***Objective:*** To determine the diagnostic performance of maternal abdominal visceral adipose tissue thickness, measured by ultrasound, in predicting gestational diabetes mellitus (GDM). ***Patients and methods:*** A prospective diagnostic study was conducted on low-risk pregnant women attending our antenatal care clinic. All underwent abdominal visceral adipose tissue (VAT) measurement by two-dimension transabdominal ultrasound twice, at late first trimester (gestational age: GA 11–14 weeks) and second trimester (GA 18–22 weeks). All patients underwent a two-step approach for screening and diagnosis of GDM between GA 24 and 28 weeks. ***Results:*** A total of 141 women were recruited into the study; including 32 (22.7%) women with GDM, and 109 (77.3%) women of non-GDM, between GA 24 and 28 weeks. The means VAT at the 1st, 2nd trimester and the difference of VAT of GDM group were 4.0 ± 0.27 cm, 5.7 ± 1.12 cm, and 1.6 ± 0.91 cm respectively. The means VAT at 1st, 2nd trimester and the difference of VAT of non-GDM group were 3.8 ± 1.01 cm, 5.4 ± 1.07 cm, and 1.6 ± 1.12 cm respectively. There were no significant differences of VAT measurements (1st, 2nd and the difference) between both groups. The VAT thickness was slightly greater in the GDM group but the mean differences between 1st and 2nd trimester were comparable between the two groups. The diagnostic performance of VAT, maternal age and body mass index (BMI) in predicting GDM was comparable. ***Conclusion:*** Measurement of maternal visceral adipose thickness in early pregnancy is not effective in predicting GDM among Thai women, which is different from most studies conducted on western women. However, a trend of higher VAT in the GDM group was noted.

## 1. Introduction

Gestational diabetes mellitus (GDM) is a common obstetric complication with a prevalence of about 8–24.5% [1]. GDM is characterized by insulin resistance, induced by placental hormones. It can adversely impact both maternal and fetal outcomes. In the affected pregnancies, the mothers have an increased risk of pregnancy-induced hypertension (PIH), cesarean delivery, and postpartum hemorrhage, whereas the neonates have a higher risk of macrosomia and its associated morbidities such as shoulder dystocia, hypoglycemia, and other related conditions [2]. Additionally, mothers with GDM have an increased risk of type 2 diabetes mellitus within five years after birth [3]. As the incidence of GDM rises, it is necessary to identify pregnant women at risk and early diagnosis of GDM. Screening and diagnosis are recommended at GA 24–28 weeks for low/average-risk pregnancies and at first visit or early gestation for high-risk pregnancies to detect undiagnosed type 2 diabetes mellitus (DM) [4].

Several risk factors can be used for screening for GDM. Obesity is considered a distinct and influential risk factor for development of type 2 DM and GDM. Therefore, body mass index (BMI), as a parameter of overweight and obese status, is commonly used to define risk of GDM [5]. However, BMI is reflective of general adiposity, while adipose tissue distribution may differ among women with the same BMI [6]. Insulin resistance, dyslipidemia, and high serum levels of free fatty acids, which are associated with GDM, are more closely related to visceral adiposity or central obesity than overall BMI [7]. Fat distribution in the abdominal region is mainly confined to the subcutaneous and intra-abdominal areas. This intra-abdominal fat is associated with an increase in inflammatory cytokines, metabolic disease, and insulin resistance, which increases the risk for GDM [8]. Martin et al. [9] demonstrated that a larger depth of visceral adipose tissue (VAT) in the first trimester is associated with hyperglycemia at 24–28 weeks gestation.

The pathophysiology of diseases related to central fat involves three mechanisms [8]. Firstly, there is an excessive release of free fatty acids in the liver, which triggers decompensation characterized by hyperinsulinemia, dyslipidemia, and hyperglycemia. Secondly, visceral adipocytes can release visfatin, a cytokine that is linked to diabetes and cardiovascular disease. Lastly, visceral adiposity can activate the hypothalamus pituitary-adrenal axis to induce sympathetic over-activity. Maternal visceral fat thickness, as quantified by ultrasonography, is associated with increased fasting glucose [10], impaired glucose tolerance test, pre-eclampsia, and preterm delivery [11,12]. However, importantly, most previous studies focused separately on a specific group of BMI (non-obese, overweight, or obese), whereas central adiposity is strongly associated with metabolic syndrome and cardiovascular disease regardless of obesity status. Several studies have demonstrated that measurement of abdominal VAT depth by ultrasonography during early pregnancy can be a potential predictor for several health conditions in late pregnancy, including glucose intolerance, insulin resistance, metabolic syndrome, large-for-date newborns, and GDM. Recently, Thaware et al. [13] showed that ultrasound measurement of VAT in early pregnancy can possibly improve sensitivity of selective screening for GDM, which, compared with the universal oral glucose tolerance test (OGTT), is likely to reduce by half the numbers requiring this test. However, further studies are now required for confirmation. Also, though meta-analyses show that VAT can be used to assess the occurrence of GDM, external validation studies are recommended [14,15]. To date, it is still unclear whether ultrasonography-measured VAT can improve the detection rate of GDM, when compared with simply using pre-pregnancy BMI. Moreover, few studies focus on Asian women, who have different body shapes that might affect the performance of VAT in predicting GDM. Accordingly, we conducted this study, which aimed to evaluate the performance of VAT measurement in the first and second trimesters in predicting subsequent GDM.

## 2. Patients and Methods

This study is a single-center, prospective cohort study, conducted on low-risk pregnant women who attended antenatal care at Maharaj Nakorn Chiang Mai Hospital, Thailand, between March 2022 and February 2023. All participants were consecutively counseled and enrolled with written informed consent, following the 1975 Declaration of Helsinki on Ethical Principles for Medical Research Involving Human Subjects. This research was conducted with ethical approval from the Institutional Review Board (Research Ethics Committee 4; Faculty of Medicine, Chiang Mai University (Study code: OBG-2565-08872, Date of Approval 30 March 2022). The participants were prospectively enrolled. The inclusion criteria were (1) singleton pregnancy, (2) maternal age of 18 years or more, (3) low-risk pregnancy, and (4) gestational age of 11–14 weeks, based on the reliable last menstrual period and sonographic fetal biometry in the first trimester. The exclusion criteria included (1) underlying medical diseases such as pre-gestational DM or chronic hypertension, (2) corticosteroids or anti-glycemic drug users, (3) early GDM, diagnosed prior to 20 weeks of gestation, and (4) fetal abnormalities.

Data Collection: The baseline characteristics and clinical data were recorded at the time of recruitment, including maternal age, parity, gestational age at VAT measurement, and obstetric outcomes. The ultrasound study for VAT measurements was performed twice, at 11–14 weeks and 18–22 weeks of gestation. All participants underwent a two-step approach for diagnosis of GDM at GA for 24–28 weeks using the National Diabetes Data Group criteria, as follows: a 50-g oral glucose challenge test (GCT) was firstly tested regardless of the fasting status. If the plasma glucose level at one hour was 140 mg/dL or less, it was interpreted as negative and non-GDM was diagnosed. If the glucose level was more than 140 mg/dL, then a 100-g oral glucose tolerance test (OGTT) was performed. The plasma glucose was measured at fasting, 1-, 2-, and 3-h after 100-g loading. The cut-off values for diagnosis were: fasting ≥ 105 mg/dL, 1 h ≥ 190 mg/dL, 2 h ≥ 165 mg/dL, and 3 h ≥ 145 mg/dL. A diagnosis of GDM was made if at least two values exceeded the cut-off. If these criteria were not met, non-GDM was diagnosed. After delivery, pregnancy outcomes were assessed and recorded shortly after birth. The primary outcome was diagnosis of GDM or non-GDM. The secondary outcomes included fetal birth weight, large-for-date fetuses, and other obstetric complications. The participants were categorized into two groups: (1) pregnant women who developed GDM and (2) pregnant women who did not develop GDM.

**Figure 1:**
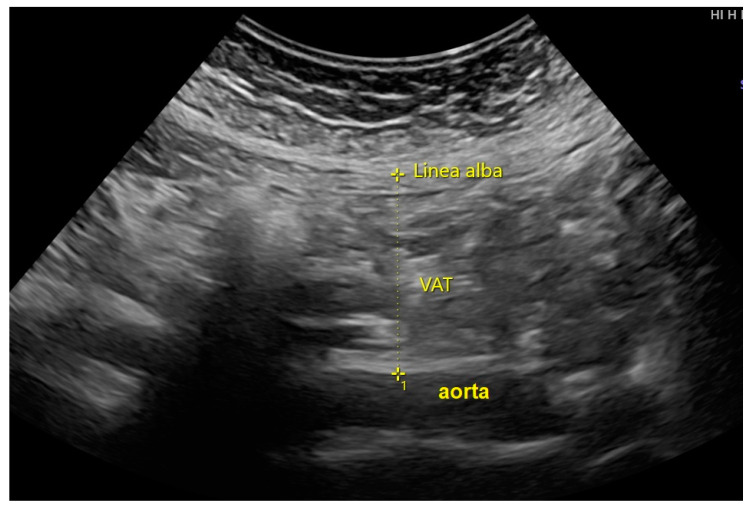
The measurement of visceral adipose tissue (VAT) at the abdominal wall, on the mid-sagittal plane.

*VAT measurement:* An ultrasound examination was performed by the authors and maternal-fetal medicine specialists using a Hitachi Aloka F37 model (Hitachi Aloka Medical Ltd.; Wallingford, CT, USA), equipped with a transabdominal curvilinear transducer and 3.5–5 MHz frequency. On examination, the two-dimensional ultrasound transducer was placed on the maternal abdomen approximately 2 cm above the umbilicus, in the xipho-umbilical line, mid-sagittal plane along with linear alba. The transducer was finely adjusted to keep the ultrasound beam exactly vertical and perpendicular to the skin and the image was then optimized to clearly visualize the posterior aspect of the junction of the two rectus abdominis muscles (the linear alba) and the anterior aspect of the abdominal aorta. The examination was performed without compression pressure, which could have altered the thickness and shape of the body layer. To prevent being influenced by maternal breathing activity or abdominal wall tightness, all frozen images were taken just immediately after expiration. The thickness of visceral fat was measured between linear alba to the anterior wall of the aorta as shown in Figure 1. The average of the three best measurements was used to estimate VAT.

*Statistical analysis:* Data analyses were performed using the statistical package for the social sciences (SPSS) software, version 26.0 (IBM Corp. Released 2019. IBM SPSS Statistics for Windows, Version 26.0 Armonk, NY, USA: IBM Corp). The continuous data were presented as mean ± SD for the data with normal or median (interquartile range: IQR) for non-normal distribution. To compare the continuous data between the GDM and non-GDM group, Student’s *T*-test or Mann–Whitney U was used, whereas Chi-square was used for comparison of the categorical variables. A *p*-value of less than 0.05 was considered statistically significant. A receiver operated characteristics (ROC) curve was used to determine the diagnostic performance of VAT measurement in predicting GDM among all participants.

## 3. Results

One hundred and forty-one cases were enrolled, and two were lost to follow up for the outcomes. The remaining 139 cases were available for analysis. In total, 31 cases developed GDM, and 108 cases did not develop GDM. The prevalence of GDM was 22.7%. The mean maternal age and the mean BMI were significantly higher in the GDM group than those in the non-GDM group (32.4 ± 4.83 Kg/m^2^ vs. 30.3 ± 5.10 Kg/m^2^, *p*-value 0.046 and 24.1 ± 5.77 Kg/m^2^ vs. 22.3 ± 3.65 Kg/m^2^, *p*-value 0.042, respectively). The gestational age at delivery in the GDM group was significantly earlier than the non-GDM group. The other baseline characteristics were comparable between both groups, as presented in Table 1.

**Figure 2:**
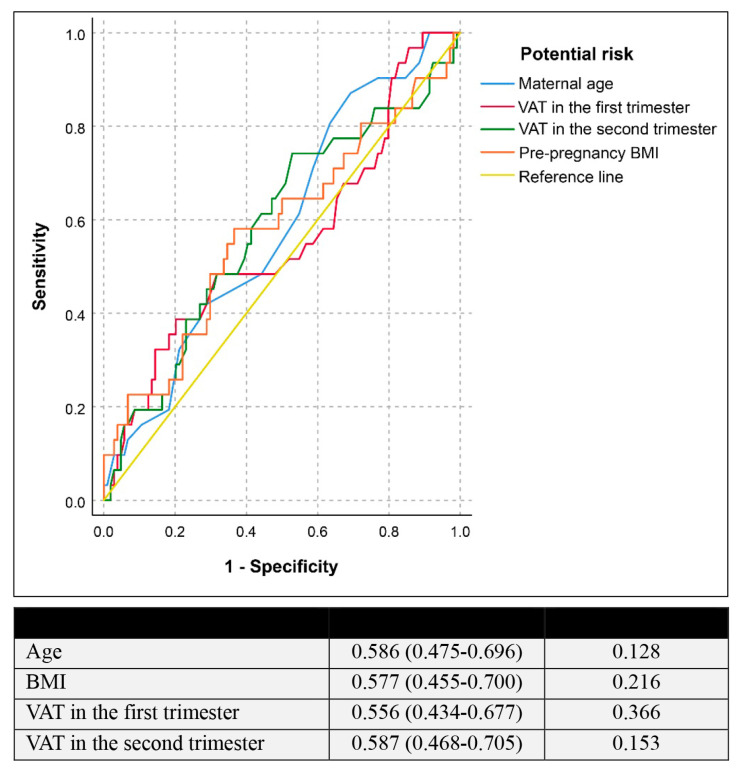
Receiver operating characteristic curve of the potential risk factors in predicting gestational diabetes mellitus (Null hypothesis: true area = 0.5).

Table 2 represents the correlation between VAT and the development of GDM. There was no significant correlation between VAT and GDM. However, VAT thickening was slightly increased in the GDM group, when compared to that in the non-GDM group, but did not reach the statistically significant level. The VAT differences from the first and second are similar in GDM and non-GDM groups (1.6 ± 0.91 vs. 1.6 ± 1.12, respectively; *p*-value: 0.926). Based on the receiver operating characteristic (ROC) curve, as presented in Figure 2, VAT measurement in both trimesters was a poor screening test in predicting GDM, providing the area under the ROC of only 0.556 (95% CI: 0.434–0.677) and 0.587 (95% CI: 0.434–0.677) for VAT, measured in the first and second trimester, respectively. If a cut-off of 4.5 cm for VAT in the first trimester was used, the test gave a sensitivity of 37.5% with a specificity of 80.6%. The analysis of intra-observer variations of VAT measurements in the first and second trimester showed high reliability with intra-class correlations of 0.958 (95% CI: 0.944–0.968) and 0.960 (95% CI: 0.948–0.970), respectively.

Concerning potential risk factors for the development of GDM, maternal age and pre-pregnancy BMI were significantly associated with an increased risk of GDM on univariate analysis while only maternal age was still significant on multivariate analysis, as presented in Table 3. VAT values both in the first and the second trimester were not significantly associated with an increased risk of GDM on both univariate and multivariate analysis.

## 4. Discussion

The insight gained from this study is that VAT thickness both in the first and second trimester tends to be higher in GDM groups than that in non-GDM groups but not significantly different, whereas BMI in GDM groups is significantly higher. The diagnostic performance of VAT in both trimesters in predicting the occurrence of GDM among the Thai population is relatively poor. This is different from most previous studies which were conducted on western pregnant women [9,11,13,16,17,18,19,20,21,22].

As already known, obesity or increased BMI, especially abdominal fat, carries a higher risk of GDM. The link between adipose tissue and GDM is biologically plausible as follows [23,24,25,26]. Though the true pathogenesis is unclear, a number of adipocytokines certainly play a role, especially adiponectin and leptin. Leptin levels are positively associated with insulin resistance while adiponectin has potent insulin-sensitizing effects and enhances insulin secretion. During pregnancy, there is a significant increase in leptin and decrease in adiponectin when compared with non-pregnancy. Accordingly, pregnancy potentiates insulin resistance, resulting in GDM. Additionally, adiponectin levels are lower in the first or second trimester of pregnancy among women who later develop GDM than non-GDM women, whereas leptin levels are higher.

BMI and VAT may be the same risk factor for GDM, both representing the body fat component, though VAT was more closely associated with either GDM or metabolic syndrome in most previous studies [13,16,27,28]. However, this study indicates that the diagnostic performance of VAT is not clearly superior to BMI. Though adiposity or VAT assessment is theoretically helpful in predicting GDM, our results suggest that BMI is preferred to VAT assessment since BMI measurement is simple and reliable with no need of any effort; whereas VAT, though relatively simple, needs technical practice. Also, the reproducibility and clinical benefit is still unclear. Therefore, our results do not support adding VAT measurement to our conventional screening test for GDM in clinical practice.

Since the increased visceral adipose tissue, directly reflective of central obesity, which is associated with adverse outcomes mentioned above, is more likely associated with metabolic disorders than overall obesity and theoretically more likely associated with development of GDM, several attempts have been made to measure abdominal fat by ultrasound to predict GDM. The correlation between abdominal fat thickness, measured in the first and second trimester, and subsequent development of GDM has been studied in many studies [9,11,13,16,17,18,19,20,21,22,28,29,30,31]. Most studies demonstrated a significant association of increased VAT in early pregnancy and increased risk of GDM in late pregnancy. However, the magnitude of risk is markedly varied with an odds ratio from 1 to 34, from slightly better than BMI in Zhang’s study in China to extremely better in D’Ambrosi’s study in Italy, whereas one study in Brazil demonstrated that VAT depth measured in the first half of pregnancy is not better than pre-pregnancy BMI in predicting insulin resistance and related biochemical measures in later pregnancy [30]. Nevertheless, the results of those studies could not be perfectly compared with high reliability because of the racial factor which highly impacts on GDM. Most studies were conducted on western populations, such as Canada [9,11,16], England [13,17,18], Italy [19], Turkey [20,21,22], and Brazil [28,30]. Very few studies were conducted on Australian [31] or Asian (China) populations [29]. Additionally, diagnosis of GDM was based on different criteria, most using a one-step approach [13,16,28,29] with 75 g-OGTT or International Association of Diabetes and Pregnancy Study Group (IADPSG) criteria [32], and two-step approach [9,11,21,22], as preferred by American College of Obstetricians and Gynecologists [4].

This study evaluated the performance of VAT in predicting GDM among pregnant women in Northern Thailand, probably representing the Asian population. VAT thickness in the first or second trimester was not significantly different between the GDM and non-GDM groups. The incidence of GDM in this study was as high as about 22.7%, similar to a prevalence of 22% in a very recent study in our population [33]. VAT in the GDM group is slightly increased, when compared with that in the non-GDM group. Martin et al. [9] reported a mean VAT of 4.0 + 1.4 cm in the first trimester of a normal pregnancy, and a cut-off of 4.74 cm was associated with abnormal GCT, consistent with other reports which showed a positive correlation between VAT and GDM, suggesting it might be a better predictor for GDM than BMI [10,19,21]. Also, Rocha et al. [28] reported a VAT cut-off of 4.5 cm (OR 13.4, 95% CI 2.9–61) in predicting GDM. Benevides et al. [34] performed the validity test for cut-off of 4.5 cm and showed 81% sensitivity and 41% specificity for GDM. In our study, the mean VAT in the GDM group is 4.0 cm, which is in the normal range for Martin’s research [9] and lower than the cut-off (4.5 cm). If we used 4.5 cm as a cut-off to predict GDM, the sensitivity would be as low as 37.5%. In this study, the AUC of ROC also shows a poor correlation in predicting GDM. Moreover, the VAT difference between the first and second trimester or magnitude of the increase was similar in both groups. The GDM group did not have an abnormal increase of visceral adipose tissue. However, the pre-pregnancy or early pregnancy VAT value is correlated with BMI.

The reason why effectiveness of VAT depth in this study was not as good as that reported in most previous studies is unclear. However, we hypothesize that racial or genetic factors might be responsible for the difference. Most previous studies were conducted on pregnant western women [9,10,11,13,19,21,22,30]. In comparison, the women’s BMI and VAT depth in such studies were obviously greater than those in our study, while the prevalence of GDM was relatively high in our study: 22.8% in universal screening. These differences might explain the difference in diagnostic performance of VAT depth, as described below:

Importantly, in this study, pre-pregnancy BMI and VAT depth of Thai pregnant women were lower than those noted in most previous studies. Note that the means of VAT depth in the first trimester were 3.8 cm and 4.0 cm in the GDM and non-GDM group in this study, less than those in the studies reported by Alves et al. [16] (5.2 cm and 6.3 cm) and Pontual et al. [30] (mean VAT 6.9 cm). Likewise, the means of pre-pregnancy BMI were 22.3 Kg/m^2^ and 24.1 Kg/m^2^ in non-GDM and GDM group in this study, less than those in the studies reported by Alves et al. [16] (25.5 Kg/m^2^ and 26 Kg/m^2^), Bourdages et al. [11] (24.8 Kg/m^2^ and 26.7 Kg/m^2^), D’Ambrosi et al. [19] (24.7 Kg/m^2^ and 26.8 Kg/m^2^), and De Souza et al. [10] (mean 25.1 Kg/m^2^), etc. Interestingly, although pre-pregnancy BMI and VAT in both trimesters were lower among Thai pregnant women, the prevalence of GDM was much higher. The prevalence of GDM was 22.7% in this study, compared to less than 10% in most western studies [4]. Note that the criteria for diagnosis of GDM in this study were based on the National Diabetes Data Group (NDDG) criteria, which has a higher cut-off than that in Carpenter and Coustan guideline and 75 g-OGTT as recommended by the International Association of Diabetes and Pregnancy Study Groups (IADPSG) [32], used in many studies. This implies that if we had used a one-step approach or 75 g-OGTT, or used the criteria of Carpenter and Coustan, the prevalence would have been much higher than 22.7%. In fact, our previous study showed that the prevalence of GDM in Thai population was as high as 32.0% with the one-step approach or 75 g-OGTT [35]. The fact mentioned above emphasizes that, different from the western pregnant women, BMI or VAT among Thai or Asian pregnant women might be a less potential risk factor for GDM, when compared to the impact of racial factors. Accordingly, BMI and VAT are possibly less sensitive in predicting subsequent development of GDM, because obesity and increased VAT depth are responsible for only a small portion of pregnant women with GDM among Thai or Asian women. In other words, though pre-pregnancy BMI as well as VAT is a risk factor for GDM in Thai populations, a large portion of Thai women with GDM are not obese and could not be explained by increased BMI or VAT depth.

The strengths of this study are as follows: (1) The prospective nature of the study with the VAT measurements prior to the diagnosis of GDM might provide more reliability of the results. (2) High homogeneity of the study population in terms of ethnicity, reducing the confounding effects from mixed racial factors found in many studies. The limitations include: (1) The sample size was potentially too small to gain enough power to show a significance of a small VAT difference, if it existed, between both groups. (2) Inter-observer variation, which is more likely reflective of reproducibility in actual practice, was not evaluated, especially the measurement in the second trimester, which is more challenging, associated with anatomical changes caused by uterine size. Note that the fixed method of measurement was used in both trimesters, as performed in most studies. (3) Since GDM is ethnicity-dependent, our results may not suitably be applied to some other ethnic groups. Thus, when compared among studies, our results should be interpreted with caution. Genetic or racial factors must be taken into consideration. The study population certainly affected the results. Also, criteria in diagnosis of GDM and screening policy were different among various studies. For example, this study uses the NDDG criteria, which has a higher cut-off than Carpenter and Coustan and 75 g-OGTT as recommended by the International Association of Diabetes and Pregnancy Study Groups (IADPSG), used in many studies.

## 5. Conclusions

Measurement of maternal VAT in early pregnancy is not effective in predicting GDM among Thai women and not superior to BMI, different from most studies conducted on western women. However, a trend of higher VAT in the GDM group was noted. The role of racial factors, which could not be predicted by VAT measurement, may play a more pronounced role in development of GDM than adipose tissue distribution. Adding VAT measurement to conventional screening tests is unlikely to increase screening performance.

## Figures and Tables

**Table 1:** Comparisons of maternal baseline characteristics and clinical data between the GDM and non-GDM groups.

Characteristics	GDM (N = 31)	Non-GDM (N = 108)	*p*-Value
Maternal			
Age (years, mean ± SD)	32.4 ± 4.83	30.3 ± 5.10	0.046 *
Multiparity (n,%)	9 (29)	40 (37)	0.523 ^#^
Weight (Kg, mean ± SD)	60.9 ± 17.90	56.2 ± 10.13	0.063 *
Body mass index (Kg/m^2^, mean ± SD)	24.1 ± 5.77	22.3 ± 3.65	0.042 *
Previous GDM (n,%)	2 (6.3)	3 (2.8)	0.318 ^#^
Previous PIH (n,%)	0 (0)	1 (0.9)	1.000 ^#^
Family history of DM	2 (6.3)	6 (5.5)	1.000 ^#^
Obstetrics outcomes			
Gestational age at delivery (weeks, mean ± SD)	37.8 ± 1.89	38.5 ± 1.37	0.029 *
Birth weight (gm, mean ± SD)	2932 ± 402.2	3029 ± 402.3	0.240 *
Blood loss (mL, mean ± SD)	279 ± 196.2	317 ± 179.1	0.324 *

SD, standard deviation; GDM, gestational diabetes mellitus; PIH, pregnancy-induced hypertension; DM, diabetes mellitus; * Student’s *T*-test; # Chi-square test.

**Table 2:** Comparison of visceral adipose tissue thickness of the study groups.

Outcomes	GDM (N = 31)	Non-GDM (N = 108)	*p*-Value *
CRL at first scan (cm: mean ± SD)	6.0 ± 0.91	5.9 ± 0.91	0.513
VAT at first scan (cm: mean ± SD)	4.0 ± 1.09	3.8 ± 1.01	0.177
BPD at second scan (cm: mean ± SD)	4.6 ± 0.27	4.6 ± 0.32	0.877
VAT at second scan (cm: mean ± SD)	5.7 ± 1.19	5.4 ± 1.07	0.188
VAT difference (cm: mean ± SD)	1.6 ± 0.91	1.6 ± 1.12	0.925

CRL, crown rump length; VAT, visceral adipose tissue; BPD, biparietal diameter; * Student’s *T*-test.

**Table 3:** Univariate and multivariate (logistics regression) analysis of various risk factors for gestational diabetes mellitus.

Risk Factors	Univariate Analysis		Multivariate Analysis	
	*p*-Value	Odds Ratio (95% CI)	*p*-Value	Odds Ratio (95% CI)
Age (year)	0.049	0.92 (0.85–1.00)	0.035	0.91 (0.82–0.99)
Pre-pregnancy BMI (Kg/m^2^)	0.048	0.91 (0.84–1.00)	0.136	0.91 (0.80–1.03)
VAT in the first trimester (cm)	0.179	0.78 (0.54–1.12)	0.728	1.10 (0.66–1.83)
VAT in the second trimester (cm)	0.188	0.78 (0.55–1.13)	0.868	0.96 (0.60–1.54)
VAT difference (cm)	0.925	0.98 (0.68–1.42)	-	-
Parity	0.412	1.44 (0.60–3.43)	0.152	0.49 (0.19–1.30)
GDM in previous pregnancy	0.360	0.42 (0.07–2.66)	0.312	0.37 (0.05–2.55)

## Data Availability

The datasets analyzed in the current study are available from the corresponding author upon reasonable request.

## Abbreviations

BMI: Body mass index; DM: Diabetes mellitus; GA: Gestational age; GCT: glucose challenge test; GDM: Gestational diabetes mellitus; IADPSG: International Association of Diabetes and Pregnancy Study Groups; NDDG: National Diabetes Data group; OGTT: Oral glucose tolerance test; PIH: Pregnancy-induced hypertension; ROC: Receiver operated characteristics; VAT: Visceral adipose tissue.
